# qSOFA Score Is Useful to Assess Disease Severity in Patients With Heart Failure in the Setting of a Heart Failure Unit (HFU)

**DOI:** 10.3389/fcvm.2020.574768

**Published:** 2020-10-28

**Authors:** Tobias Wagner, Christoph Sinning, Jonas Haumann, Christina Magnussen, Stefan Blankenberg, Hermann Reichenspurner, Hanno Grahn

**Affiliations:** ^1^Department of Cardiology, University Heart and Vascular Center Hamburg, Hamburg, Germany; ^2^Department of Cardiovascular Surgery, University Heart and Vascular Center Hamburg, Hamburg, Germany

**Keywords:** heart failiure, SOFA, qSOFA, SIRS, acute heart failure (AHF), intermediate care, heart failure care

## Abstract

**Aims:** There is no gold standard to predict outcome in acute decompensated heart failure (ADHF). Several scores for mortality prediction of patients with ADHF have been developed and mostly consist of complex regression models. None of these models has been widely adopted by clinicians. The quick SOFA score (qSOFA) is a simple score including three parameters (systolic blood pressure ≤ 100 mmHg, respiratory rate ≥22 breathes/min, and GCS <15) and is validated for discrimination of mortality risk in septic patients. Here, we adapted qSOFA score to patients admitted to a Heart Failure Unit (HFU) and assessed the prognostic accuracy.

**Methods and Results:** qSOFA, SOFA score, and SIRS criteria were assessed at admission. Clinical, laboratory, and echocardiographic parameters were recorded. A follow-up was performed 30 days after discharge. Primary outcome was all-cause mortality or readmission to hospital due do worsening of heart failure symptoms. Of 240 patients (73% male, 16–93 years), 25 patients (10%) had a qSOFA ≥2 points and 126 patients (53%) fulfilled none of qSOFA criteria. Within 30 days, the primary endpoint occurred in 46 patients (19%). Seventeen patients (7%) died and 34 patients (14%) were readmitted to hospital due to worsening heart failure. Patients with qSOFA ≥2 reached this endpoint more frequently (48 vs. 19%, *p* = 0.002), had more often dyspnea NYHA III-IV (OR 2.4, *p* = 0.005) and a higher risk for multi organ failure during hospital stay (28 vs. 9%, *P* = 0.005).

**Conclusions:** qSOFA is useful to identify patients with heart failure at high risk for worse outcome and to operationalize severity of decompensation.

## Introduction

Hospitalization for acute decompensated HF (ADHF) both *de novo* and worsening HF is related to high all-cause mortality. Furthermore, prognosis after discharge is poor but highly variable depending on several risk factors. For example, Cheng et al. observe mortality in the total cohort of 8.8% at 30 days and 36.3% at 1 year in a cohort of patients, including both HF with preserved left ventricular ejection fraction (HFpEF) and reduced LVEF (HFrEF) (1).

Therefore, tools for easy and rapid risk stratification are needed. Although some risk models exist for patients with chronic heart failure (CHF), it is unclear whether these scores can be directly applied to patients with ADHF (2–7). To be clinically meaningful, risk stratification must incorporate several clinical parameters. However, with the use of numerous parameters, such as those in the Meta-analysis Global Group in Chronic Heart Failure (MAGGIC) score or The Eplerenone in Mild Patients Hospitalization and Survival Study in Heart Failure trial (EMPHASIS-HF) risk score, more accurate risk assessment is possible, but these scores cannot be calculated rapidly without a computer (6–8).

Both Sequential Organ Failure Assessment (SOFA) and quick SOFA (qSOFA) scores are consistently associated with outcomes in septic patients (8, 9) in whom they especially discriminate mortality risk (10).

Sepsis and AHF share several clinical features, such as hypotension, delirium, and arrhythmia. These parallels inspire the translation of SOFA and qSOFA score from sepsis to ADHF. The qSOFA score contains three parameters that are frequently altered in ADHF: low blood pressure as a sign of cardiogenic shock, high respiratory rate in the course of pulmonary congestion, and altered Glasgow Coma Scale as a hallmark of hypoperfusion.

The major goal of our study is to assess the usefulness of qSOFA score for risk prediction in patients admitted to a heart failure unit (HFU). Furthermore, we aim to compare the predictive power of the qSOFA score with the SOFA score and SIRS criteria and to identify which of the scores best improves clinical decision making and assessment of severity of ADHF and patients' outcome.

## Methods

### Study Design

This was a retrospective study of all patients with suspected AHF admitted to a 16-bed HFU at Hamburg University Heart and Vascular Center between June 2017 and December 2017. Due to the retrospective and observational nature of the study, informed consent was waived. Data were retrieved from the digital patients' record. The following clinical data were collected from the day of admission: age, gender, body mass index (BMI), cause of admission, cardiovascular risk factors, NOHRIA class, first-day SOFA score (SOFA), quick SOFA score (qSOFA), SIRS criteria, Glasgow Coma Scale (GCS), mean arterial pressure (MAP) at arrival, medication, and presence of cardiogenic shock (defined by reduced LV function, need for vasopressors, systolic blood pressure <90 mmHg).

Furthermore, the following parameters were collected throughout the hospital stay: length of stay at the HFU, antibiotic therapy, need for renal replacement therapy, inotropic or vasopressor support, and mortality.

A set of laboratory parameters was measured at admission and at the second day of stay. In addition, standard echocardiography was available in the majority of patients.

A clinical follow-up was routinely performed 30 days after discharge. Parameters included NYHA class, clinical history, particular worsening of heart failure symptoms as a patient-reported outcome, hospital admission due to worsening of heart failure or other cardiovascular cause, and self-assessed clinical frailty scale.

### Scores and Definitions

We calculated qSOFA, SOFA, and SIRS scores based on physiological and laboratory data that were collected upon admission to the HFU. Standard criteria were applied with a threshold of 2 or more points for each scoring system. The baseline SOFA score was assumed zero for patients without a known preexisting organ dysfunction.

We defined end-organ dysfunction/injury from the measurements collected at baseline: (a) heart failure was defined by reduced ejection fraction (EF <50%) or NT-proBNP >750 ng/L in the case of normal EF. If no initial NT-proBNP was available, a TnT level above the upper reference limit (UR, >14 pg/nL) in context of an eGFR >60 mL/h (12) or TnT >300 pg/nL if GFR <60 mL/h was used as a marker.

(b) Renal dysfunction was defined as an eGFR <60 mL/min/1.73 m^2^ calculated using the Modification of Diet in Renal Disease equation (13, 14). (c) Liver injury/dysfunction was recognized when at least one of the following abnormal liver function tests were found: AST/ALT >3 times the UR (>150 and >150 IU/L for AST and ALT, respectively), bilirubin above the UR (>1.2 mg/mL) (15). The assessment is illustrated in Supplemental Table 1. According to the definition, single, dual, and triple organ failure could be differentiated.

#### Quick Sequential Organ Failure Assessment (qSOFA)

Quick Sequential Organ Failure Assessment (qSOFA) was defined as a categorical variable range (0–3 points), and the score was calculated according to the following three parameters: systolic blood pressure ≤ 100 mmHg, respiratory rate ≥22 breaths/min, and GCS <15 (16).

#### Systemic Inflammatory Response Syndrome (SIRS)

Criteria (range, 0–4) included temperature >38 or <36°C, heart rate >90 bpm, respiratory rate >20/min or P_a_CO_2_ <32 mmHg, leukocytes >12 or <4 /nL (17).

#### NOHRIA Classification (Stevenson Classification)

The hemodynamic situation of acute heart failure and its clinical application was classified into four groups according to Nohria et al. (11): NOHRIA A (no evidence of congestion or hypoperfusion; warm and dry), NOHRIA B (congestion with adequate perfusion; warm and wet), NOHRIA C (congestion and hypoperfusion; wet-cold), and NOHRIA L (hypoperfusion without congestion; dry-cold).

#### Frailty

Frailty was assessed using the Clinical Frailty Scale (CFS) that ranges from 1 (very fit) to 9 (terminally ill). The CFS results were categorized into five groups as follows: non-frail (CFS 1-3), vulnerable (CFS 4), mildly frail (CFS 5), moderately frail (CFS 6), and severely frail (CFS ≥7) (18).

#### SOFA Score

The severity of the disorder in any of the six vital organs of respiratory, coagulation, cardiovascular and circulatory, liver, central nervous system and renal, were scored on a 0–4 scale based on definitions of SOFA scoring system (19). Calculation of the score for each organ is summarized in Supplemental Table 2.

When calculating the full SOFA score, the PaO_2_/FiO_2_ ratio was used if arterial blood gas analysis was available, and the SpO_2_/FiO_2_ ratio was used if there was no PaO_2_ information as proposed (20). Because no mechanical ventilated patients were included, FiO_2_ was derived from oxygen insufflation (FiO_2_ = 0.21 + ([O_2_ L/min] ^*^ 0.04).

### Outcomes

The primary outcome was all-cause mortality or readmission to hospital due do worsening of heart failure symptoms. Further, NYHA class, worsening of heart failure symptoms without readmission, and CFS were assessed as outcome parameters at follow-up. Worsening of heart failure symptoms was measured as a patient-reported outcome. Patients were asked about changes in dyspnea, edema, exertion intolerance, angina pectoris, palpitations, and dizziness.

### Statistical Analysis

For all variables, descriptive statistics were computed. Depending on the scale of measure, data are presented as numbers and percentages, means and standard deviations (SD), medians and interquartile ranges, or proportions with 95% confidence intervals (CI).

For comparison between groups, exclusively non-parametric tests were used. Continuous variables were compared between groups by using the Mann-Whitney *U*-tests and Kruskal-Wallis tests in case of interval-scaled variables. Fisher's exact test was used to compare categorical variables between the groups. Correlation analysis was performed with Spearman's rank test.

The predictive performances of the SOFA, qSOFA, and SIRS scores were analyzed with receiver operating characteristic (ROC) curves and area under the ROC curve (AUC) values.

Data were analyzed with IBM SPSS version 24 for Microsoft Windows. Two-tailed tests of significance were considered to be significant at a *p* < 0.05 and highly significant at *p* < 0.01.

## Results

### Baseline Characteristics

Two hundred forty patients (mean age 63 years, ±15 years SD) are included. Herein, 64 are female (26.7%). Two thirds of patients (63.3%) are classified as NOHRIA A, one third as NOHRIA B, and a minority of 2.1 and 1.7% as NOHRIA C and L, respectively. About half of patients had preexisting CHF (49.2%). Heart, renal, and liver failure according to the abovementioned definitions are quite frequent. Half of patients (47.5%) are affected by failure of two of these organs. Triple organ failure is present in 16.7% and chronic kidney disease (CKD) in 25.6% (Table 1).

**Table 1:** Clinical and demographic baseline data.

**Demographic baseline data**	
Age (mean, SD)	62.8 (15.5)
Females (*n*, %)	64 (26.7)
BMI (mean, SD)	26.8 (5.3)
**CLINICAL BASELINE DATA**	
▪ Need for O_2_ insufflation	99 (41.7)
▪ MAP <70 mmHg at arrival or vasopressor therapy	47 (19.6)
▪ Suspected infection (pneumonia, infective endocarditis, sepsis)	25 (10.4)
**RISK FACTORS**	
▪ Arterial hypertension	125 (52.1)
▪ Nicotine	84 (35.0)
▪ Diabetes mellitus	56 (23.3)
▪ Dyslipidemia	62 (25.8)
**CO-MORBIDITIES**	
▪ CHF	119 (49.2)
▪ COPD	17 (7.0)
▪ CKD	62 (25.6)
▪ Neoplasm	27 (11.2)
▪ Liver cirrhosis	6 (2.5)
**NOHRIA CLASSIFICATION (STEVENSON CLASSIFICATION)**	
▪ A (warm/dry)	152 (63.3)
▪ B (warm/wet)	79 (32.9)
▪ C (cold/dry)	5 (2.1)
▪ L (cold/wet)	4 (1.7)
**THERAPY AT ADMISSION**	
▪ Antibiotic therapy	105 (43.7)
▪ Need for hemodialysis	19 (7.9)
▪ Inotropic and vasopressor support	24 (10.0)
**ORGAN FAILURE AT ADMISSION**^a^	
▪ Renal failure	120 (50.0)
▪ Heart failure	191 (79.6)
▪ Liver failure	81 (33.8)

Data are prepared as n (%) if not indicated differently.

a *criteria for organ failure are explained in Supplemental Table 1*.

### qSOFA, SOFA, and SIRS Criteria

**Figure 1:**
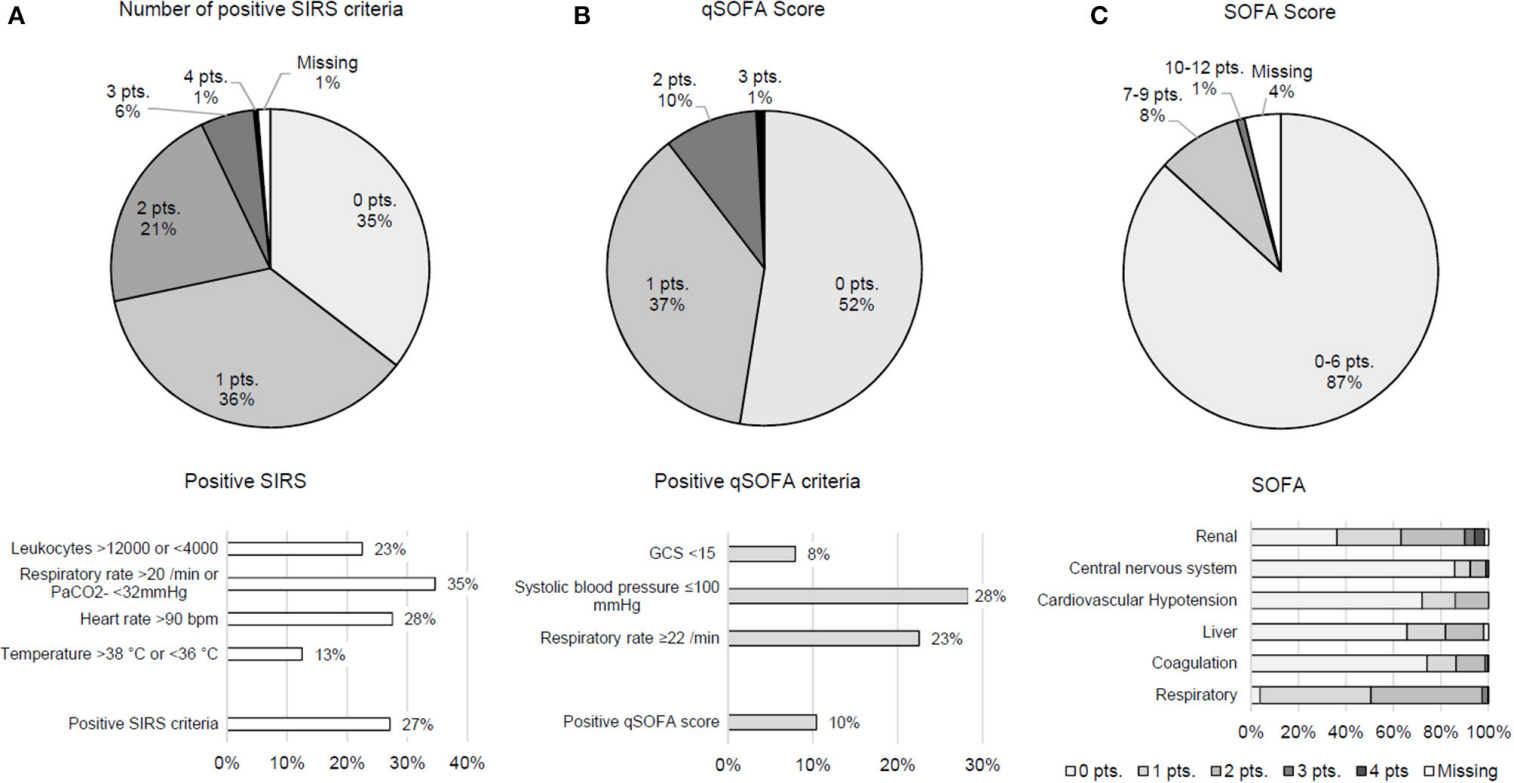
Distribution of SIRS **(A)**, qSOFA **(B)**, and SOFA score **(C)**.

Results indicate that 27.1% of patients met ≥2 SIRS criteria, and qSOFA score was positive (≥2 points) in 10.4; 47.5% of patients fulfilled at least one qSOFA criterion. Therein, hemodynamic compromise was most frequent. In the vast majority of patients (86.8%), values of the comprehensive SOFA score were unremarkable (0–6 points). Notably renal and respiratory criteria accounted for higher scores (Figure 1). Patients with a suspected infection reached higher means in SIRS (mean 0.82 ± 0.77 SD vs. 1.24 ± 1.06, *p* = 0.004), SOFA score (mean 3.02 ± 1.76 SD vs. 3.88 ±2.27, *p* = 0.006), and qSOFA score (mean 0.45 ± 0.62 SD vs. 0.84 ± 0.77, *p* < 0.001).

The comparison of selected parameters assessed during HFU stay and at follow-up shows that patients with at least one qSOFA criterion were sicker and had worse outcome parameters. These patients were more frequently affected by failure of heart, liver, and kidney at baseline (OR 1.84, *p* = 0.044) and 3–6 days after admission (OR 2.62, *p* = 0.005). They further needed more often inotropic or vasopressor support (OR 7.14, *p* < 0.001) and had an ejection fraction below 30% (OR 2.40, *p* = 0.003). In addition, a higher proportion of these patients were categorized other than NOHRIA A at arrival (OR 4.02, *p* < 0.001). At follow-up, patients with a positive qSOFA score described worsening of heart failure symptoms and especially dyspnea according to NYHA III and IV more often (OR 2.02, *p* = 0.055 and OR 2.43, *p* = 0.005, respectively; Table 2).

**Table 2:** Association of qSOFA ≥1 point with selected clinical and diagnostic parameters.

	**qSOFA 0 pt. *n* (%)**	**qSOFA 1-3 pts. *n* (%)**	**Chi^2^**	***p***	**OR**	**95% CI**
**AT HFU STAY**						
qSOFA and ≥2 organ failure at baseline	52 (45.2)	63 (66.3)	4.2	0.044	1.84	1.01–3.33
Triple organ failure at baseline	14 (14.6)	27 (29.3)	5.6	0.014	2.43	1.18–5.01
qSOFA and ≥2 organ on day 3–6	38 (48.1)	51 (70.8)	7.3	0.005	2.62	1.34–5.14
Triple organ failure on day 3–6	7 (8.9)	20 (27.8)	7.6	0.002	3.96	1.56–10.04
Ejection fraction ≤ 30%	28 (25.0)	43 (44.3)	8.7	0.003	2.40	1.34–4.30
Inotropic or vasopressor support	4 (3.2)	20 (17.5)	13.7	<0.001	7.14	2.38–21.42
NOHRIA B, C or D	28 (22.2)	60 (52.6)	23.8	<0.001	4.02	2.30–6.99
**FOLLOW UP**						
Worsening of heart failure symptoms compared to screening	17 (15.6)	24 (26.7)	3.7	0.055	2.02	1.01–4.03
Hospital admission due to heart failure symptoms	14 (12.7)	20 (22.0)	3.0	0.082	2.00	0.95–4.20
Death or Hospital admission due to heart failure	16 (14.3)	30 (29.7)	7.5	0.006	2.58	1.31–5.08
NYHA III or IV	29 (26.9)	40 (46.0)	7.7	0.005	2.43	1.34–4.41

### Outcomes

Within 30 days, 41 patients (18.6%) reported worsening of heart failure symptoms irrespective of readmission to a hospital. About one third described dyspnea NYHA III or IV. Seventeen patients (7.7%) died, and 34 patients (15.4%) were readmitted to the hospital due to worsening of heart failure symptoms. Clinical aspects and outcomes at follow-up are presented in Table 3.

**Table 3:** Clinical aspects and outcomes at follow-up.

**Clinical history at follow-up (*n* = 221)**	
Worsening of heart failure symptoms as compared to screening	41 (18.6)
Hospital admission due to worsening of heart failure	34 (15.4)
Hospital admission due to cardiovascular cause	18 (8.1)
Stroke	1 (0.5)
Myocardial infarction	2 (0.9)
Death	17 (7.7)
Need for Intervention/OP	59 (26.7)
**CLINICAL FINDINGS AT FOLLOW-UP (*****n*** **=** **221)**	
Oedema of lower extremities	58 (26.2)
Oedema of upper extremities	14 (6.3)
Ascites	8 (3.6)
Anasarca	21 (9.5)
**NYHA**	
I	78 (35.3)
II	48 (21.7)
III	52 (23.5)
IV	17 (7.7)
**CLINICAL FRAILTY SCALE (CFS)**^**a**^	
Non-frail (CFS 1-3)	90 (40.7)
Vulnerable (CFS 4)	31 (14.0)
Mildly frail (CFS 5)	25 (11.3)
Moderately frail (CFS 6)	23 (10.4)
Severely frail (CFS ≥7)	27 (12.2)

a *according to Rockwood et al. (18)*.

In total, in 46 patients (19.2%), the primary endpoint occurred. A positive qSOFA score (≥2 points) was measured in 21.7% (10 patients), and only 6.6% of patients (11 in total) who did not reach the primary endpoint had a positive qSOFA (*p* = 0.002) at admission.

**Figure 2:**
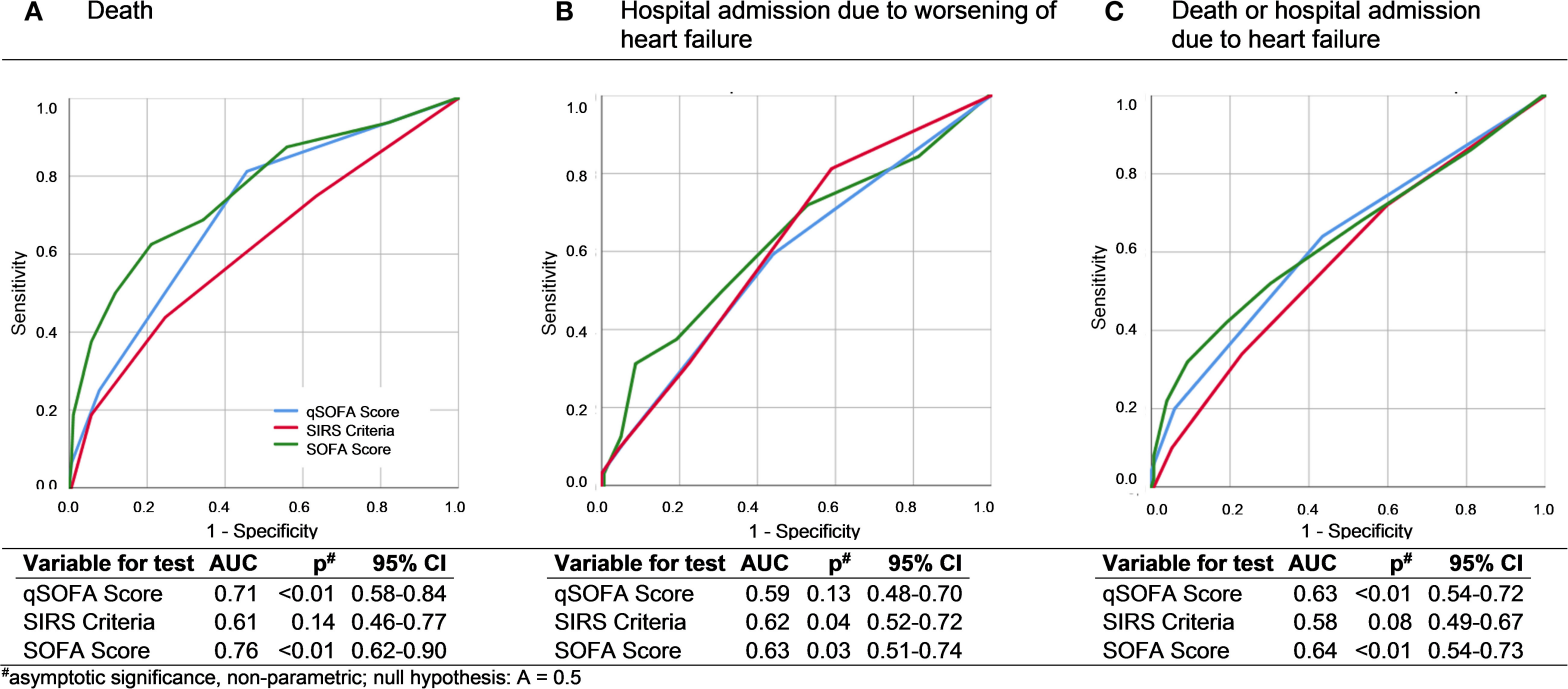
ROC curves and AUC analysis of qSOFA, SIRS, and SOFA for death **(A)**, hospital admission due to worsening of heart failure **(B)**, and a combined endpoint of death and worsening of heart failure symptoms **(C)**.

The ROC analysis of qSOFA (AUC 0.63, 95% CI 0.54–0.72, *p* < 0.01), SOFA (AUC 0.64, 95% CI 0.55–0.74, *p* < 0.01), and SIRS criteria (AUC 0.58, 95% CI 0.49–0.67, *p* = 0.09) show no fundamental differences regarding the prediction of the primary endpoint death, readmission to hospital due to heart failure symptoms, and a combination of both (Figure 2).

Regarding this primary endpoint, the 1-point cutoff of qSOFA score was chosen because of a higher Youden index (0.209 vs. 0.151, sensitivity 66%, specificity 44%). Thus, a qSOFA score on this note (≥1 points) was present in 65.2% (30 patients) who reached the primary endpoint and in 42.5% (71 patients) who did not (*p* = 0.006).

## Discussion

In this study on 240 patients admitted to our HFU, we assessed and compared the predictive value of an initial clinical assessment using the SOFA, qSOFA, and SIRS scores for death and readmission for worsening heart failure. Our patient population was chosen to present features of acute or acute on chronic heart failure. Main findings of this study are the following:

(I) Presence of heart failure at admission was assessed by an algorithm that identified 79.6% of patients. About half of patients had preexisting CHF. ADHF patients often show features of coexisting multiorgan failure. Relevant shares of patients with renal failure (50.0%) and liver failure (33.8%) demonstrate severity of disease.

(II) We could show that a positive qSOFA score at admission is associated with a worse outcome defined as death or rehospitalization because of heart failure symptoms.

(III) Further, we could show that predictive power checked by ROC did not differ between SIRS criteria, SOFA, and qSOFA score and that all three scores better predict mortality than rehospitalization.

There is no gold standard risk score to predict outcome in AHF. Particularly, mortality and readmission events are not dependent on the ejection fraction (1). Several scores for mortality prediction of patients with ADHF have been developed using clinical data collected from registries or randomized trials. Most of these attempts led to complex regression models: For instance, calculation of the MAGGIC Score (6) includes 13 variables. This score requires detailed knowledge about the patient's history (COPD, diabetes, first diagnosis of heart failure in the past 18 months) and echocardiography (ejection fraction), and assessment of some factors (age or systolic blood pressure) is dependent on ejection fraction, which complicates its use. An easier score may lack statistical precision, but complicated statistical methods are obstacles for bedside use. A comparison of seven models that predict inpatient mortality of ADHF published by Lagu et al. (21) detects similar discrimination of different clinical models designed to stratify patient risk at the bedside. However, none of these models has been widely adopted by clinicians, and some of these models are based on data older than a decade. Recently, Nakada et al. published a work investigating the A_2_B score containing age, anemia, and BNP at discharge as trichotomized parameters (22). They identify A_2_B score as a good tool for stratification of disease severity and, therefore, useful for determining prognosis. Of note, for calculation of this score, a BNP value, which is usually measured at admission, is required at discharge. The availability of BNP is often restricted, and the comparability of assays is limited and influenced by several factors, e.g., renal function. Another study shows that discharge NT-proBNP levels predict outcome similarly in HFpEF and HfrEF patients (23).

Severity scores can pursue several goals. Aside from prediction of long-term outcome, in-hospital mortality or assessment of the need to transfer a patient to an intensive care unit or to a tertiary facility with a cardiac intensive care unit can be a meaningful implication of scoring. The latter may be especially important for patients who are cared for by a non-cardiologist in rural hospitals because they may lack the clinical expertise to identify a patient with ADHF who is at high risk.

Severity of decompensation is related to the function of other organs in two different ways: On the one hand, hypoperfusion causes organ failure; on the other hand, previous preexisting organ failure can aggravate heart failure (e.g., congestion in case of oliguric renal failure), which is associated with worse outcomes in these patients. In parallel to our findings, Zymlinksi et al. (24) could recently show that, in patients with ADHF, injury of more than one end-organ identifies patients at the highest risk of poor outcomes. Their cohort in which 70% of patients suffered from decompensated chronic heart failure seems to be principally comparable to ours.

SOFA and qSOFA scores measure hallmarks of end-organ dysfunction and were originally established to describe disease severity or predict outcome in pneumonia and sepsis. In 2016, qSOFA was introduced as a bedside tool for the identification of patients at risk of sepsis outside the intensive care unit (8). SOFA is not originally defined to predict outcome but to describe the sequence of complications in distinct organs (19). However, it is considered to be useful for the prediction of outcomes of critically ill patients. In the past 3 years, a small number of studies adapt qSOFA for patient populations outside the ICU, and there are only few studies comparing the prognostic accuracy of SOFA, qSOFA, and SIRS with differing results. Song et al. find that a positive qSOFA score has high specificity outside the ICU in early detection of in-hospital mortality, acute organ dysfunction, and ICU admission but low sensitivity limiting its use as a predictive tool for adverse outcomes (25). In a meta-analysis, Liu et al. find that qSOFA shows a poor performance in predicting mortality of infected patients outside the ICU (26). However, performance of SOFA seems to be relevantly dependent on disease and patient selection: Ahnert et al. find that SOFA score can serve to score severity of community-acquired pneumonia and is proposed as an endpoint for biomarker and therapeutic studies (27). Although Costa et al. (28) find an outperformance of SOFA and qSOFA compared to SIRS in cancer patients admitted to ICU, Probst et al. (29) find that SOFA allows a better discrimination for in-hospital mortality than qSOFA or SIRS. In contrast, here we find that positive SIRS criteria as well as SOFA and qSOFA score in ADHF are associated with worse outcomes without obvious differences in discrimination.

A positive qSOFA in diseases other than sepsis has been discussed before, but the influence of qSOFA on outcomes has not been investigated yet (30). Strong association with worse outcomes let us conclude that qSOFA criteria could be part of a score replenished by other clinical features. However, limited sensitivity and specificity indicate that qSOFA score alone cannot sufficiently predict outcome in ADHF.

This study has some limitations. First, it uses retrospective data that may introduce a potential source of information bias. In addition, the information extracted from the patient record focuses on the patient's clinical state at admission. Ongoing clinical reevaluation is not considered. Second, data on long-term survival or nursing home requirement were not taken into consideration. Third, we did not consider other explanatory variables favoring occurrence of the primary endpoint, such as chronic hemodialysis or immunity status. About 18% of patients had an ICU stay prior to admission to HFU and, thus, already underwent hemodynamic optimization. However, we think that this approach represents a realistic model of the clinical population. Finally, it is an observational study set in a single center, limiting generalization of results. Further research could investigate the impact of delta scores from hospital admission to discharge or compare accuracy of qSOFA score with heart failure–specific scores like the MAGGIC score.

In conclusion, qSOFA is a useful tool to operationalize disease severity in adult in-patients with acute heart failure and can be considered when making therapeutic strategies and decisions.

## Data Availability Statement

The data analyzed in this study is subject to the following licenses/restrictions: Datasets can be provided on request. Requests to access these datasets should be directed to to.wagner@uke.de.

## Ethics Statement

Ethical review and approval was not required for the study on human participants in accordance with the local legislation and institutional requirements. Written informed consent for participation was not required for this study in accordance with the national legislation and the institutional requirements.

## Author Contributions

HG and CS conceived, designed, and supervised the study. JH, HG, and TW performed data collection. TW performed data and statistical analysis. TW and HG wrote the manuscript. All authors contributed to manuscript revision, read, and approved the submitted version.

## Conflict of Interest

The authors declare that the research was conducted in the absence of any commercial or financial relationships that could be construed as a potential conflict of interest.

## Abbreviations

ADHF: Acute decompensated heart failure
AHF: Acute heart failure
AUC: Area under the curve
BMI: Body mass index
CFS: Clinical Frailty Scale
CHF: Chronic heart failure
CKD: Chronic kidney disease
COPD: Chronic obstructive pulmonary disease
GCS: Glasgow Coma Scale
LVEF: Left ventricular ejection fraction
HFpEF: Heart failure with preserved left ventricular ejection fraction
HFrEF: Heart failure with reduced left ventricular ejection fraction
HFU: Heart Failure Unit
MAP: Mean arterial pressure
NOHRIA: Hemodynamic classifications of acute heart failure and their clinical application by Nohria et al. (11)
qSOFA: Quick Sequential (Sepsis-related) Organ Failure Assessment
ROC: Receiver operating characteristics
SIRS: Systemic Inflammatory Response Syndrome
SOFA: Sequential (Sepsis-related) Organ Failure Assessment
UR: Upper reference limit.
