# Supplementary material for: qSOFA Score Is Useful to Assess Disease Severity in Patients With Heart Failure in the Setting of a Heart Failure Unit (HFU)
Source: Front Cardiovasc Med. 2020 Oct 28;7:574768. doi: 10.3389/fcvm.2020.574768 (PMC7655543; doi:10.3389/fcvm.2020.574768)
Supplement: Supplementary file 1 [file Data_Sheet_1.docx]

Supplemental Table 1

| **Definition of organ failure** | | |
| --- | --- | --- |
| **Renal failure** | GFR<60 mL/h | |
| **Liver failure** | Bilirubin>1.2 mg/dL *or* GPT >150 U/L *or* GOT >150 U/L | |
| **Heart failure** | **Positive criteria** | **Negative criteria** |
|  | ▪ NT proBNP >750 | ▪ NT proBNP <250 (independent from EF)  ▪ NT proBNP <750 (EF normal) |
|  | ▪ Troponin >14 pg/nL if GFR >60 mL/h  ▪ Troponin >300 pg/nL if GFR <60 mL/h | ▪ Troponin ≤14 pg/nL *and* EF normal *or* EF missing |
|  | ▪ EF reduced | ▪ EF normal |

Supplemental Table 2

| **Organ/System** | **Variable** | **SOFA Score** | | | | |
| --- | --- | --- | --- | --- | --- | --- |
|  |  | **0** | **1** | **2** | **3** | **4** |
| Respiratory | PaO_2_/FiO_2_ [mmHg] or SpO_2_/FiO_2_ [%] | >400 or >476 | <400 or  <476 | <300 or <357 | <200^¶^ or <214 | <100^¶^ or <89 |
| Coagulation | Platelets [10^3^/µL] | >150 | ≤150 | ≤100 | ≤50 | ≤20 |
| Liver | Bilirubin [mg/dL]^Ɨ^ | <1.2 | 1.2-1.9 | 2.0-5.9 | 6.0-11.9 | ≥12.0 |
| Cardiovascular Hypotension |  | No hypotension | Mean arterial pressure <70 mmHg | DA ≤5 or dop (any dose)^§^ | DA >5, E ≤0.1or NE ≤0.1^§^ | DA >15, E >0.1 or NE >0.1^§^ |
| Central nervous system | GCS | 15 | 13-14 | 10-12 | 6-9 | <6 |
| Renal | Creatinine [mg/dL]^#^ or urine output [mL/d] | <1.2 | 1.2-1.9 | 2.0-3.4 | 3.5-4.9 or <500 | ≥5.0 or <200 |

NE indicates norepinephrine; DA, dopamine; E, epinephrine; and FiO_2_, fraction of inspired oxygen.

^¶^Values with respiratory support

^Ɨ^To convert bilirubin from mg/dL to µmol/L, multiply by 17.1

^§^Adrenergic agents administered for at least one hour (doses given are in µg/kg/min)

#Ton convert creatinine from mg/dL to µmol/L, multiply by 88.4.

Supplemental Table 3

| **Standard laboratory tests** | | | | | | | | |
| --- | --- | --- | --- | --- | --- | --- | --- | --- |
|  | **Baseline** | |  | | **Day 2-4** | | | |
|  | Median | IQR | 25^th^ percentile | 75^th^ percentile | Median | IQR | 25^th^ percentile | 75^th^ percentile |
| Hemoglobin [g/dl] | 11.5 | 4.0 | 9.6 | 13.6 | 10.9 | 3.8 | 9.1 | 12.9 |
| Leukocytes [Mrd/L] | 8.9 | 4.9 | 6.7 | 11.6 | 7.6 | 3.7 | 6.2 | 9.9 |
| Creatinine [mg/dl] | 1.20 | 0.91 | 0.89 | 1.80 | 1.20 | 1.00 | 0.90 | 1.90 |
| Urea day [mg/dl] | 23 | 22 | 15 | 37 | 26 | 23 | 17 | 40 |
| GOT/AST [U/L] | 37 | 54 | 21 | 75 | 33 | 36 | 20 | 56 |
| GPT/ALT [U/L] | 32 | 33 | 21 | 54 | 31 | 33 | 20 | 53 |
| Bilirubin [mg/dL] | 0.9 | 0.8 | 0.5 | 1.3 | 0.9 | 0.6 | 0.5 | 1.1 |
| Lactate [mM] | 1.1 | 0.7 | 0.9 | 1.6 | 0.7 | 0.8 | 0.5 | 1.3 |
| CRP [mg/L] | 27 | 69 | 7 | 76 | 34 | 68 | 11 | 79 |
| NT-proBNP [ng/L] | 4,554 | 10,135 | 1,456 | 1,1591 | 4,411 | 17,219 | 966 | 18,185 |
| Troponin T [pg/mL] | 141 | 1,224 | 36 | 12,600 | 398 | 1,615 | 67 | 1,682 |
